# Biomarkers in phase I–II chemoprevention trials: lessons from the NCI experience

**DOI:** 10.3332/ecancer.2015.599

**Published:** 2015-11-24

**Authors:** Eva Szabo

**Affiliations:** Lung and Upper Aerodigestive Cancer Research Group, Division of Cancer Prevention, National Cancer Institute, National Institutes of Health, 9609 Medical Centre Drive, Room 5E–102, Bethesda, MD 20892, USA

**Keywords:** biomarkers, chemoprevention, phase II clinical trials, National Cancer Institute

## Abstract

Early phase clinical trials are an essential component of chemopreventive drug development to identify signals of drug efficacy that can subsequently be explored definitively in phase III trials. Whereas phase I trials focus on safety and identification of optimal dose and schedule for cancer prevention, phase II trials focus on intermediate endpoints that are variably related to cancer development. The United States National Cancer Institute supports a programme devoted to early phase cancer prevention clinical trials. The experience, along with the benefits and limitations of the range of biomarker endpoints used in these studies, are reviewed here.

## Introduction

The development of epithelial cancer is a lengthy process that provides multiple opportunities for intervention. At the invasive disease end of the treatment spectrum, the approach to metastatic disease has undergone a paradigm shift. Molecular profiling, resulting in treatment with targeted therapies based on the presence of specific molecular abnormalities, has become a part of standard care in several diseases even as the full potential of immunotherapy is just starting to be understood [1, 2]. Even more sensitive imaging and molecular techniques are being developed to diagnose early cancers so that they can be treated more appropriately with localised modalities (e.g. surgery) prior to systemic dissemination. However, the longest phase of carcinogenesis is likely to be the premalignant phase, starting with the first initiating mutations through increasing levels of molecular and histologic abnormalities that culminate in the breach of the basement membrane and local invasion [3]. The lengthy time frame and less complex molecular profiles (at least early on) of the premalignant disease phase make it a particularly attractive target for intervention.

The Division of Cancer Prevention of the United States National Cancer Institute (DCP, NCI) sponsors a clinical trials programme focused on the early clinical development of chemopreventive interventions (NCI, DCP early phase cancer prevention clinical trials programme, http://prevention.cancer.gov/major-programs/phase-0iii-cancer-prevention). The main goal of these clinical trials is to identify a signal of efficacy which provide information for further drug development and the main focus is phase II studies. In contrast to phase II cancer treatment trials that assess tumour shrinkage or progression-free survival (PFS) that is monitored by standard of care CT scans, phase II cancer prevention trials aim to prevent the development of cancer that does not yet exist and therefore need to rely on intermediate endpoints related to the carcinogenic process. Since there is no universal validated intermediate endpoint or standard way (e.g. a ‘CT equivalent’) to assess endpoints across a variety of target organs, different study designs and different study endpoints are variably informative with regard to drug efficacy. The various classes of biomarkers utilised in the NCI experiences are reviewed here.

## Intraepithelial neoplasia as a trial endpoint

Histologic preinvasive lesions have varying potential to develop into overt malignancy characterised by invasion and eventual metastasis. Often broadly grouped together under the category of intraepithelial neoplasia (IEN), these preinvasive lesions have been described in a variety of organ sites and are a recognised intermediate in the causal pathway to cancer development in many settings [3]. More than 40 years ago, the seminal work of Saccomanno *et al* [4] demonstrated that squamous cell lung cancer develops through a series of histologically recognisable stages from mild, moderate, and severe dysplasia to carcinoma *in situ* and eventually to invasive lung cancer, a process that occurs over many years. Similar stages have been identified for a number of other organs. The presence of IEN identifies a high risk population and surveillance and removal of the IEN has become standard of care for some organs, such as the colon or the cervix. Thus IEN represents a logical target for chemoprevention trials, both to identify high-risk individuals and potentially as an endpoint to assess agent efficacy.

However, although there is general consensus that the presence of IEN is associated with a higher cancer risk, the progression to cancer is lesion-specific and can be highly variable. For example, oral leukoplakia is generally recognised as a precursor to oral cancer. The course of individual lesions is difficult to predict, with spontaneous waxing and waning (including complete resolution). The overall progression rate of dysplasia to cancer is 12.1%, with a mean time of 4.3 years to transformation according to one meta-analysis (14 studies with 922 cases) [5]. Furthermore, only approximately one-half of the cancers develop at the site of a previous leukoplakia, while the rest occur elsewhere in the oral cavity [6]. Similar issues exist for IENs in other target organs [3]. This means that successful modulation of an IEN in a chemoprevention study needs to be interpreted cautiously as a signal rather than as a definitive indicator of efficacy.

Additionally, IEN may be difficult to identify in organs such as the breast or lung because of their anatomic inaccessibility. Invasive procedures, such as random fine needle aspiration for the breast or bronchoscopy for the lung, are needed to obtain tissues that can be assessed for the presence of IEN. Lam and colleagues have performed a series of chemopreventive interventions in current and former smokers with bronchial dysplasia, with the phase IIb randomised, placebo controlled trial of an inhaled steroid, budesonide, serving as a prototype for the dysplasia endpoint study design [7]. To identify 112 subjects who were ultimately randomised to the intervention, 1040 individuals were screened for sputum atypia. Among these 562 individuals underwent bronchoscopy in whom atypia was identified,. This study underscored the high regression rate of bronchial dysplastic lesions, 46–48% of individual lesions regressed after six months and approximately 30% of participants in either arm had complete regression of all lesions. It cannot be determined whether this high rate of regression is truly spontaneous or partially represent excision of the lesions and/or productive repair of the IENs because of bronchoscopic perturbation and subsequent healing. Nevertheless, partial or total removal of the IEN because of the nature of the screening biopsy prior to intervention represents a major challenge to chemoprevention study design across all organs.

Although the bronchial dysplasia clinical trial model is feasible, it requires major resources and can only identify a signal from highly ­effective interventions. Additional lessons learnt include the need for placebo controls given the high rate of regression in both study arms. Ability to identify an IEN and to power a study to account for the natural history of the lesion including spontaneous regression, limit the use of IEN as a study endpoint for chemoprevention trials. On the other hand, given that IENs are intermediates in the causal pathways of carcinogenesis, modulation of IEN provides the strongest preliminary efficacy signal for further drug development. It may be that prevention of progression of IEN or prevention of the development of new IENs may be the most appropriate efficacy endpoint for phase II trials. The positive colorectal adenoma studies used such an approach after removal of existing adenomas [8, 9]. Unfortunately, the number of participants in such studies needs to be much larger than is feasible in most phase II settings.

## Pharmacokinetic/pharmacodynamic endpoints

The rationale for examining drug levels and biomarkers of drug effect in the tissue of interest is based on the need to know that the chemopreventive agent gets into the target organ. If drug does not penetrate into the organ, then it is difficult to understand how it could be effective for prevention of cancer arising from that organ. This type of trial is typically performed in the presurgical setting and represents a useful way to gain further understanding of drug pharmacokinetics and mechanisms of action. However, it provides limited information that can stop drug development, but not justify phase III trials. Although drug effect on the target organ is necessary, it is not sufficient to indicate that carcinogenesis has been halted. If the process targeted by the drug is not causal for cancer development, then interruption of the particular pathway can be irrelevant.

An example of this type of study is the phase II trial of genistein, a soy isoflavone, in patients with bladder cancer scheduled to undergo surgery published by Messing and colleagues [10]. Two doses of genistein, 300 mg per day or 600 mg per day, were compared with ­placebo given for 14–21 days to 60 bladder cancer patients. The primary endpoint was pharmacodynamic, inhibition of phosphorylation of the epidermal growth factor receptor (EGFR). A statistically significant effect was demonstrated for the lower, but not higher dose in cancer tissue, and also not in the adjacent normal tissue. Other secondary endpoints were not modulated. These data suggest a bimodal effect whereby lower doses are more effective than higher doses, which is consistent with other studies in the literature [10]. The short intervention period would not be expected to significantly impact the existing tumour or any premalignant lesions and hence no effect on tumour could be seen. Given that it is not well established that EGFR signalling is critical for bladder carcinogenesis and given that it is not known whether the amount of inhibition of EGFR observed in this study is biologically sufficient to prevent growth, these results do not provide enough evidence for a subsequent phase III effort. The results do help in the selection of the lower dose for future smaller studies.

## Cancer-associated biomarkers as endpoints

A variety of biomarkers known to be deregulated in cancer have been examined in chemoprevention trials, sometimes in the context of cancer or IEN, or else in histologically normal tissues. The rationale is based on the recognition that many of these processes become deregulated early during carcinogenesis and thus modulation should be an indicator of agent activity. Since IENs are uncommon and frequently difficult to identify, the ability to perform analysis on normal tissues simplifies the endpoint assessment. However, interpretation of such studies may be difficult. If a biomarker is not critical to carcinogenesis, modulation may not be meaningful. There may be multiple pathways to carcinogenesis operating in a target organ, so modulation of just one pathway may be insufficient to prevent the development of cancer via other (or bypass) pathways. Interpreting the effect of marker modulation in the histologically normal albeit at-risk epithelium is difficult to correlate with prevention, and it is particularly difficult to determine how much modulation is actually necessary to produce a biologically significant effect.

The marker that has been studied most frequently across multiple target organs is the proliferation index, Ki-67. An example of effective use of this biomarker is a phase II comparison of topical 4-hydroxytamoxifen (4-OHT) and oral tamoxifen in women undergoing surgery for ductal carcinoma *in situ* (DCIS) [11]. The goal of this study was to determine if topical 4-OHT was as effective as oral tamoxifen but with fewer systemic side effects. Ki-67 has been extensively studied in neoadjuvant endocrine treatments for breast cancer and is generally accepted as a valid intermediate endpoint in this setting (although not necessarily for other classes of agents) [12]. A six to ten weeks of topical 4–OHT or oral tamoxifen equivalently reduced Ki-67 by 3.4% and 5.1%, respectively (P ≤ 0.03 in both, between-group P = 0.99), while endocrine and coagulation biomarker effects were reduced in the 4-OHT arm compared with tamoxifen. In the context of comparison of the test agent (topical 4-OHT) with an agent known to be effective (tamoxifen) and known to reliably affect the target biomarker (Ki-67), modulation of Ki-67 was highly informative and provided support for later phase drug development.

An alternative approach to biomarker development in early phase trials is exemplified by the use of gene expression signatures from normal bronchial brushings, used to study the effects of chemopreventive interventions. Gustafson *et al* [13] showed that a phosphatidylinositol 3-kinase (PI3K) gene signature was upregulated in the normal bronchial epithelium of smokers with lung cancer or bronchial dysplasia, indicating that this pathway activation occurs early during carcinogenesis. Using samples collected from participants in a small chemoprevention trial, they further showed that the PI3K activation signature was reversed in subjects who responded to the drug myo-inositol. *In vitro* studies showed that myo-inositol, a precursor of phosphatidylinositol, inhibited PI3K activity [13]. These results need to be validated in a larger study (which has recently been completed but not yet reported), but they have important implications regarding molecular criteria for cohort selection and endpoint biomarkers. It is substantially easier to obtain brushings of normal bronchial mucosa than to identify and biopsy bronchial dysplasia. If indeed PI3K activation identifies smokers at particularly high risk for cancer, then it could be used to select the highest risk cohort that stands to benefit the most from interventions. It may also serve as an appropriate intermediate endpoint for clinical trials.

A clinical trial model that uses high throughput analyses such as gene expression signatures before and after treatment could potentially use smaller numbers of participants and a shorter duration of the intervention. This data-intensive approach also has the potential to identify mechanisms of action of the intervention and to identify smaller panels of biomarkers that could subsequently be adapted to more routine clinical use.

## Imaging endpoints in chemoprevention trials

Anatomic areas that are beyond the reach of endoscopes, such as the peripheral lung, are particularly difficult to study as tissues are not easily obtainable. Various imaging modalities offer the opportunity to monitor response non-invasively, which would simplify the conduct of chemoprevention trials in comparison to the use of invasive biopsies to monitor molecular endpoints as discussed above. However, ­anatomic imaging such as CT or mammography does not image most IENs well and cannot discern the identity of the imaged lesions. In the absence of biopsies, it is difficult to correlate the observed changes with underlying biology.

The introduction of helical CT, as used for CT screening has allowed the visualisation of the peripheral lung. Even with the caveats listed above, this allows the assessment of the effects of chemopreventive interventions on the lung compartment that gives rise to adenocarcinoma, which is the most common histologic subtype of lung cancer. Veronesi *et al* studied the effects of inhaled budesonide on ­CT-detected indeterminate lung nodules in a phase IIb clinical trial [14]. Within the context of a CT screening trial, participants with persistent ­CT-detected peripheral lung nodules were randomised to inhaled budesonide or placebo for a year (the same treatment regimen as studied by Lam *et al* described above [7]). Although the overall response across all nodule types did not differ between budesonide or placebo, a trend toward regression was noted in the non-solid (ground glass) lesions. With extended follow-up for four additional years after treatment ­cessation, the decrease in lesion size in the non-solid lesions became significant [15]. On the other hand, there was no change in the size of persistent solid nodules that had not grown in the year prior to study entry.

This clinical trial is the first lung cancer prevention trial to focus on the peripheral lung; the type of nodule that should be the focus of the trial was not intuitively obvious during study design. The natural history of ground glass opacities is not well understood. Older studies suggest that approximately 25–60% of ground glass opacities that are surgically resected represent atypical adenomatous hyperplasia which is felt to be a precursor of lung adenocarcinoma [16, 17]. Long term follow-up of small non-calcified nodules in the National Lung Screening Trial showed that long-term cancer risk was significantly increased in persons with ground-glass opacities, consistent with the hypothesis that these lesions are lung cancer precursors [18]. Taken together with these data, the experience with different nodule types allows for refinement of the clinical trial model. A current study assessing aspirin in the same CT-screened population is focusing only on non-solid or partially-solid persistent nodules (NCT02169271).

## Conclusions

Selection of biomarker endpoints for early phase chemoprevention clinical trials is driven by a balance between feasibility and the amount of information to be obtained from modulation of that biomarker. The more causally linked the biomarker is to cancer development, the more informative it is with regard to subsequent drug development. Thus, the majority of trials sponsored by the NCI assess biomarkers from tissues obtained through invasive procedures. Although non-invasive biomarker assessment, such as by imaging, is particularly attractive because it facilitates accrual, it provides limited information due to the potential heterogeneity of the signal being imaged and thus potential to dilute out any efficacy signal. The more distantly related a biomarker is to cancer causation, the greater the potential to dilute out the efficacy signal.

There are many unanswered questions regarding optimal usage of biomarkers in clinical trials. It is unclear how much of a response is needed to provide clinical benefit, especially when trials are designed to assess the effect of the intervention on a specific marker such as the proliferative index or a signalling intermediate. The timing of the intervention during the process of carcinogenesis is critical. The question arising now is, is it reasonable to expect an agent to be effective in regressing existing lesions (e.g. IENs) rather than preventing initiation or progression of existing lesions? In the context of colorectal cancer prevention, the most effective chemopreventive interventions studied to date actually prevented the occurrence of new adenomas after colonoscopic removal of existing adenomas [8, 9]. These issues do not have easy resolution. Nevertheless, a deeper understanding of the molecular pathways leading to cancer development in various target organs and the development of better model systems to identify effective agents and their mechanisms of action are critical to making progress in chemoprevention. Translation to clinical trials, with refinement of the clinical trial models as allowed by technologic advances, helps to identify a pathway forward.

## Conflicts of interest

None.

## Author contributions

Eva Szabo was responsible for the conception, writing, and approval of this paper.
